# Parkinson’s disease model in zebrafish using intraperitoneal MPTP injection

**DOI:** 10.3389/fnins.2023.1236049

**Published:** 2023-08-25

**Authors:** Noor Azzizah Omar, Jaya Kumar, Seong Lin Teoh

**Affiliations:** ^1^Department of Anatomy, Faculty of Medicine, Universiti Kebangsaan Malaysia, Kuala Lumpur, Malaysia; ^2^Department of Medical Sciences, Faculty of Medicine and Health Sciences, Universiti Sains Islam Malaysia, Bandar Baru Nilai, Malaysia; ^3^Department of Physiology, Faculty of Medicine, Universiti Kebangsaan Malaysia, Kuala Lumpur, Malaysia

**Keywords:** neurodegenerative disease, *Danio rerio*, tyrosine hydroxylase, dopaminergic neuron, neurotoxin

## Abstract

**Introduction:**

Parkinson’s disease (PD) is the second most common neurodegenerative disease that severely affects the quality of life of patients and their family members. Exposure to 1-Methyl-4-phenyl-1,2,3,6-tetrahydropyridine (MPTP) has been shown to reflect behavioral, molecular, and proteomic features of PD. This study aimed to assess the protocol for inducing PD following MPTP injection in adult zebrafish.

**Methods:**

Fish were injected with 100 μg/g of MPTP intraperitoneally once or twice and then assessed on days 1 to 30 post-injection.

**Results:**

Between one-time and two-time injections, there was no significant difference in most locomotor parameters, expressions of *tyrosine hydroxylase-2* (*th2*) and *dopamine transporter* (*dat*) genes, and dopaminergic neurons (tyrosine hydroxylase positive, TH+ cells) counts. However, caspase-3 levels significantly differed between one- and two-time injections on the day 1 assessment.

**Discussion:**

Over a 30-day period, the parameters showed significant differences in swimming speed, total distance traveled, *tyrosine hydroxylase-1* (*th1*) and dat gene expressions, caspase-3 and glutathione protein levels, and TH+ cell counts. Days 3 and 5 showed the most changes compared to the control. In conclusion, a one-time injection of MPTP with delayed assessment on days 3 to 5 is a good PD model for animal studies.

## 1. Introduction

Parkinson’s disease (PD) is the second most common neurodegenerative disease, characterized by a spectrum of motor rigidity, bradykinesia, tremor, rigidity, and postural instability (Tysnes and Storstein, 2017). This occurs as a result of the formation of Lewy bodies, which eventually leads to the loss of dopaminergic neurons in the substantia nigra pars compacta (Kalia and Lang, 2015). It has been established that the prevalence of PD peaked in the older age group (Pringsheim et al., 2014; Williams-Gray and Worth, 2016). In this aging population era, PD significantly impacts the healthcare system due to its chronic pattern of illness and its psychosocial impact on caregivers (García-Ramos et al., 2016). In view of the fact that there is still no cure, the current treatment regime faces challenges involving drug tolerance and other side effects. Studies have been vastly designed to find the pathogenesis and potential cure of this disease. Animal studies have been one of the methods widely used over the decades, where animals are used to mimic PD for further investigation. The two primary methods for inducing PD in animal models include either neurotoxin chemical induction or a transgenic gene modification approach. Neurotoxins such as 6-hydroxydopamine (6-OHDA), 1-methyl-4-phenyl-1,2,3,6-tetrahydropyridine (MPTP), rotenone, paraquat, maneb, and trichloroethylene have been widely used in animal models (Vijayanathan et al., 2017; Liu et al., 2018; Zeng et al., 2018; Cao et al., 2019).

Zebrafish (*Danio rerio*) is a freshwater fish belonging to the Cyprinidae of the order Crypriniformes. Their natural habitat is in Southeast Asian streams, rivers, or well-vegetated pools (Spence et al., 2008). There have been a growing number of studies using zebrafish as an animal model due to its high fecundability, ease of maintenance, and similarity to the human genome. Its females can spawn every 2–3 days, with a short generation time of 3–4 months (Kimmel et al., 1995). Their external fertilization results in relatively large transparent eggs, making them accessible for manipulation and monitoring through all developmental stages (Kimmel et al., 1995). The development stage starts from the embryonic pre-hatching phase at 0–72 h post fertilization (hpf); larvae stage at 1 to 29 days post fertilization (dpf); juvenile fish 90 dpf to 2 years; and the aging phase from 2 to 5 years (Kalueff et al., 2014). Their development is rather rapid, with the precursors to all major organs developing within 5 dpf (Kimmel et al., 1995). It has been reported that 70% of human genes have at least one zebrafish orthologue. Mutations in these genes will result in pathologies similar to those in humans (Howe et al., 2013). In the early phase of zebrafish, studies have shown a variety of human disease conditions were able to be replicated in a zebrafish model (Barut and Zon, 2000). Henceforth, the swift adoption of zebrafish as an animal model for the past three decades was assisted by technological advances including late targeted mutagenesis (Burton, 2015). This has been reflected in many diseases, including neurological diseases. Although there are differences in complexity between zebrafish and the human nervous system, there is evidence of the conservation of critical structures and molecular components that shows its potential as a suitable animal model to study the basic pathogenesis of neurological diseases (Burton, 2015). However, there is still relatively little data on the use of neurotoxins to induce PD (Razali et al., 2021). The challenges faced in handling the zebrafish model include the alteration of dose from other animal models, the delivery method, and the timing of assessment before the regenerative capability of their nervous system (Bhattarai et al., 2016, 2020; Vijayanathan et al., 2017).

MPTP is a type of meperidine analog formed as a by-product of the synthesis of 1-methyl-4-phenyl-4-propionoxypiperidine (MPP), synthetic heroin that is 5–10 times more potent than morphine (Ziering and Lee, 1947; Langston, 2017). The earliest case of Parkinsonism induced by a meperidine analog was reported by Davis et al. (1979), where they found a Parkinsonism feature in a young man who is an avid drug user of various drugs, including a home-synthesized unknown meperidine analog. It was not until 1983 that MPTP was discovered and linked to Parkinsonism feature. A group of neurologists and scientists has found a series of patients that develop Parkinsonism features as early as 4 days following intravenous injection of the “new synthetic heroin,” which was later found to be MPTP (Langston et al., 1983). Following that, an eruption of studies evolved to understand and analyze this chemical and its role in causing Parkinsonism.

MPTP readily crosses the blood–brain barrier, and induces Parkinson-like behavioral, molecular, and proteomic features in various animal models, including zebrafish (Sarath Babu et al., 2016; Beaudry and Huot, 2020; Mustapha and Mat Taib, 2021; Sun et al., 2022). However, most studies involving zebrafish have induced PD using MPTP in the embryonic or larval stage (Lam et al., 2005; McKinley et al., 2005; Ustundag et al., 2020; Zhao et al., 2020; Cansiz et al., 2021). There is a sparse study on using MPTP for the PD model in adult zebrafish. To the best of our knowledge, there have only been five studies on MPTP in adult zebrafish with different dosing, administration routes, and assessment periods (Anichtchik et al., 2004; Sarath Babu et al., 2016; Saszik and Smith, 2018; Selvaraj et al., 2019; Kalyn and Ekker, 2021; Table 1). These studies have shown promising results in terms of locomotor analysis, dopaminergic gene dysregulation, dopamine (DA) protein depletion, and significant changes in tissue section analysis. However, questions arise about the suitable dose, the need for multiple doses, or the timing of assessment post-injection. Hence, this study aims to design a structured protocol for the PD zebrafish model using MPTP and to assess the effectiveness of this method via an assessment of the locomotor effects, dopaminergic gene expression, protein analysis, and tissue section analysis.

**Table 1:** Summary of previous studies that used MPTP as a neurotoxin in the adult zebrafish PD model.

Study	MPTP dose	Route of administration	Frequency of administration	Time of assessment	Findings that mimic PD				Other findings
					Locomotor	Gene dysregulation	Proteomic analysis	Tissue changes	
Sarath Babu et al. (2016)	50 μg/fish	Intraperitoneal	One-time injection and two-time injection	24 h after injection	Increased freezing bouts and distance traveled in two-times injection group	13 significant dysregulation of gene markers in 2-time injection and 8 genes in 1-time injection	TH protein showed no significant changes in both groups, PARK8 protein significant in both group	Overexpression of synuclein in the optic tectum, significant down-regulation of TH+ cell in 2x injection group	A dose above 75 μg/fish resulted in casualty. Two-dose does not affect casualty. MPTP-injected fish showed erratic swimming patterns.
Anichtchik et al. (2004)	20 μg/g	Intramuscular	One	24, 72, 144, 216 h after injection	Total distance and speed reduced from day 1 after injection and slowly recovered by day 9 after injection	–	Maximum reduction in DA level observed 2 days after MPTP injection No changes in TH level, caspase 3	No significant changes in TH+ neuron counts and caspase-3 stains No significant change in DNA fragmentation TUNEL staining	–
Selvaraj et al. (2019)	100 μg/g	Intraperitoneal	One	24 h after injection	Reduced locomotion, distance traveled, and speed with longer freezing duration	Down-regulation of *dat* gene in the MPTP group	Significant decrease in DA level in HPLC analysis		–
Saszik and Smith (2018)	2 mmol/L for 2 min in 300 mL beaker	Water immersion	One	Following MPTP exposure	No significant changes in total distance and speed of swimming	–	–	–	A low dose of MPTP altered social shoaling swim behavior
Kalyn and Ekker (2021)	25 mM for 4 consecutive days	Cerebroventricular microinjection	Four	Following 4^th^ injection	Reduced total distance in swimming and speed, increase in freezing activity following 3^rd^ and 4^th^ injection	*dat* and *th1* gene down-regulated in the MPTP group	–	Dopaminergic neurons were affected in optic bulb, telencephalon, and periventricular pretectal nucleus. Significant increase in fragmented mitochondria in the MPTP group	The concentration of 35 mM and higher resulted in casualties in fish *sox2* and *nestin* gene expression up-regulated after day 4 suggesting immediate regenerative activation All swimming patterns and histological changes return to normal within 2 weeks

## 2. Materials and methods

### 2.1. The animals

**Figure 1:**
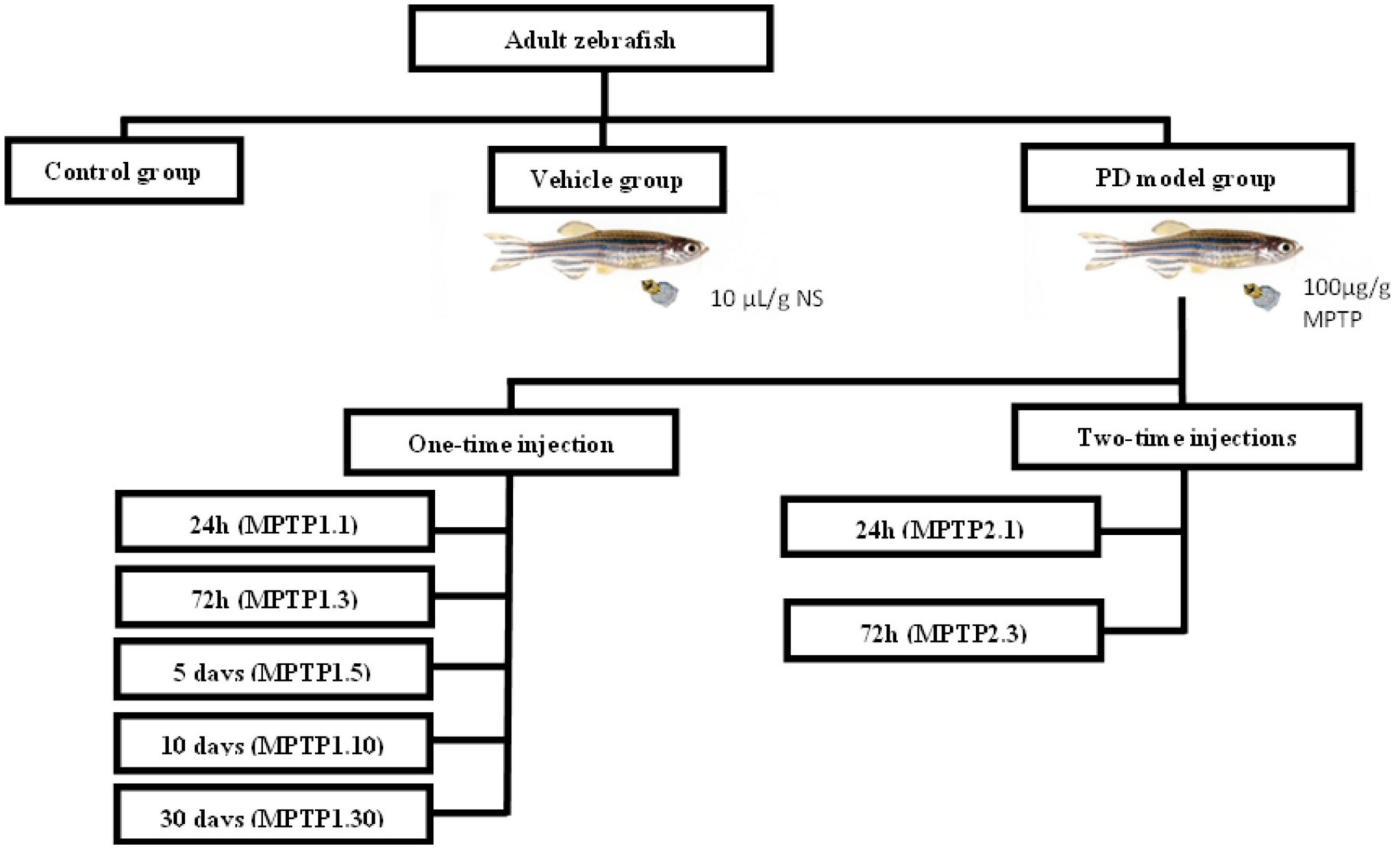
Grouping of experimental animals. Fish were divided into control, vehicle, and MPTP groups. The MPTP1 group received one injection and was further divided based on assessment day, namely 24 h post-injection (MPTP1.1), 72 h post-injection (MPTP1.3), day 5 assessment (MPTP1.5), day 10 assessment (MPTP1.10) and day 30 assessment (MPTP1.30). MPTP2 group received the second dose 24 h after the first dose and was assessed either at 24 h post-second injection (MPTP2.1) or 72 h post-second injection (MPTP2.3). Assessment on all intervals includes a neuro-behavioral assessment from locomotor activity, gene expression, protein level, and histochemical analysis.

Adult zebrafish aged 4–6 months were housed in 6 L freshwater aquaria at a density of 3–5 fish per L (Cachat et al., 2010) with the temperature kept at 27 ± 0.5°C and a controlled normal photo regimen (14 h light and 10 h dark). The fish were fed adult zebrafish food twice daily. Experiments were conducted after 2 weeks of acclimatizing to laboratory conditions (Howe et al., 2013; Teoh et al., 2015). The sample size was calculated using Power and Sample size calculation (PPS) version 3.1.6, October 2018 by William D. Dupont and Walton D Plummer, Jr. with α score of 0.05, power of 0.8, and a m score of 1, based on the study conducted on the assessment of zebrafish as a PD model (Sarath Babu et al., 2016; Vijayanathan et al., 2017). The sample size for locomotor behavior was found to be *n* = 4 fish per group, and for gene expression is *n* = 6 per group. On the objectives that had no previous study, such as each of the protein level markers and dopaminergic neuron cell counts, a pilot study with a sample size of 6 per group per test was conducted, adapting the Markov Chain Monte Carlo approach (Allgoewer and Mayer, 2017). To provide a standardized representation, each group and each test were kept at *n* = 6 of fish as the numbers provided in the PPS calculator are within this number. The fish were grouped into Control, Vehicle, and MPTP groups. The control group was not injected with any solution, and the vehicle group was injected with saline (10 μL/g weight) intraperitoneally to mimic the volume injected in the treatment group. MPTP groups were divided into one- and two-time injection groups with assessment intervals (Figure 1).

### 2.2. PD model induction

Before the procedure, fish were fasted for 24 h by withholding feeding and placing 3 layers of marbles at the base of the fish tank to ensure they were on an empty stomach. MPTP solution (Cat#M0896, Sigma, United States) was diluted in saline to make a 10 μg/μL solution (Selvaraj et al., 2019; Mustapha and Mat Taib, 2021). Each fish was weighed individually by transferring the fish to a beaker filled with 1/3rd of the system water. The dose of MPTP was calculated at 100 μg/g per fish. The fish’s weight ranged from 0.440 to 0.760 g. The volume of the solution was kept at 10 μL/g (Turner et al., 2011a,b; Al Shoyaib et al., 2019). Following dose measurement, the fish were transferred one at a time to a beaker with 0.0035% benzocaine for the anesthetizing process. Upon reaching anesthesia stage 3 (Coyle et al., 2004), the fish was transferred to a slitted surgical bed, belly up. The desired dose of MPTP was administered intraperitoneally using a 30G needle with a Hamilton syringe into the midline between the pelvic fins (Kinkel et al., 2010). Once MPTP was administered, the fish was placed in a beaker with 1/3rd of the system water for recovery, and then transferred to the fish tank according to the groups.

In the first part of the study, we assessed the differences between one- and two-time injections of MPTP. The MPTP1 group received only one injection and was further divided into two groups, assessed 24 h (MPTP1.1) and 72 h post-injection (MPTP1.3). The MPTP2 group received the second dose 24 h after the first dose and was assessed either at 24 h (MPTP2.1) or 72 h post-second injection (MPTP2.3). In the second part of the study, we have extended the assessment up to 30 days post-injection in light of the study conducted by Anichtchik et al. (2004) and Kalyn and Ekker (2021) where they found reversibility of MPTP in zebrafish on the second week post-injection. Based on the preliminary data between the one- and two-time injection groups, we have concluded to focus on the reversibility effect of the one-time injection group only. Hence, the assessments were extended at intervals of 5 (MPTP1.5), 10 (MPTP1.10), and 30 days post-injection (MPTP1.30) for the one-time injection group. On the day of assessment, the fish were assessed for their locomotor behavior, dopaminergic gene expression, protein level, and immunohistochemistry analysis.

### 2.3. Locomotor assessment

The locomotor assessment was conducted by placing the fish in a 2.5 L system water in a tank. Video recording of the fish swimming pattern was performed by c992 pro stream webcam LogiCapture and analyzed using the SMART tracking device Smart 3.0.02, Panlab Harvard Apparatus^®^. The fish were assessed individually by placing one fish at a time in the locomotor tank. The fish were allowed to acclimatize to the new tank for 5 min before the recording, followed by an assessment of their total swimming distance, speed of swimming, time spent on the top half of the tank, and latency to reach the top half of the tank for 5 min (Cachat et al., 2010).

### 2.4. Gene expression analysis

The sequence of the zebrafish gene was retrieved from the National Centre for Biotechnology Information Database. Chromosomal gene location was identified using UCSC Genome Browser,^1^ and Ensembl Genome Browser.^2^ The dopaminergic genes that were assessed are namely tyrosine hydroxylase (*th1*, *th2*), and DA transporter (*dat*) (Table 2). The fish were euthanized using ice water immersion kept at 0 to 2°C. Their brains were dissected and homogenized using 400 μL of TRIZOL reagent, and RNA was extracted as per the manufacturer’s protocol. Following that, the 500 ng RNA sample was converted to cDNA in a 20 μL reaction volume using the reverse transcriptase kit (Protoscript^®^ First Strand DNA Synthesis Kit) following the manufacturer’s protocol. The cDNA samples were mixed with the desired primers (Table 2) and proceeded with real-time PCR (Luna^®^ Universal qPCR Master Mix). The β actin1 (*actb1*) gene was used as a reference gene, and data analysis was based on the relative expression of genes using the formula 2-ΔΔCq.

**Table 2:** Oligo sequence for real-time PCR.

Gene		Oligo sequence (5′→3′)	Product size	Accession number
*th1*	Forward	TGGATCAGGATCACCCAGGA	149 bp	NM_131149.1
	Reverse	GTAGACCTCCCGCCATGTTC		
*th2*	Forward	GAATGCCACATGGGAGGTTT	129 bp	NM_001001829.1
	Reverse	AGCTGAGGGATCTGGTCTTCT		
*dat*	Forward	GAGTCGGGTTTGGTGTGCTA	71 bp	NM_131755.1
	Reverse	GGCGTCTCTGTAGCAGTTGT		
*actb1*	Forward	GCCTTCCTTCCTGGGTATGG	78 bp	NM_131031.1
	Reverse	ATGTCCACGTCGCACTTCAT		

### 2.5. Protein expression analysis

The expression of DA, glutathione S Transferase (GST), and caspase-3 (CASP3) enzymes and brain-derived neurotrophic factor (BDNF) were assessed using a zebrafish enzyme-linked immunosorbent assay kit (ELK Biotechnology). Brain samples (0.006–0.007 g each) were homogenized using cold Phosphate Buffered Solution (PBS) in a 1:9 (weight: volume) dilution. The homogenates were centrifuged for 5 min at 10,000 g at 4°C. Around 50–100 μL supernatant was collected to be used on each well as per the manufacturer’s protocol. In each run, the samples were processed with a set of freshly prepared standard solutions to develop the standard curve for analysis. The samples and standard were processed in pre-coated wells with biotinylated antibodies, streptavidin-horseradish peroxidase (HRP), and 3,3′,5,5′-Tetramethylbenzidine (TMB) solution, with a series of washings with washing buffer in between. Following the administration of the stop reagent after incubation of the TMB solution, the photometric assessment was conducted as soon as possible at 450 nm and 540 nm wavelength. The optical density (OD) reading of 450 nm was deducted from a 540 nm reading to minimize error. Following that, the OD of all the wells was deducted from the mean OD of the blank. The ODs of the standard solution were plotted on a graph with a linear equation. The results of the samples were analyzed based on the plotted graph.

### 2.6. Immunohistochemistry tissue section

Fresh brain tissues were fixed in 4% PFA for 6 h and kept in a 20% sucrose solution overnight for cryoprotection. The tissue samples were frozen in Tissue-Tek^®^ Optimal Cutting Temperature compound and cryosectioned at −20°C at 14 μm thickness. The tissue slides were stored at −80°C before the staining protocol. Tissue sections were processed using avidin-biotin and peroxidase methodologies using a Peroxidase kit for Mouse Primary Antibody (Dako ARK^™^, Cat#K3954, Agilent, United States). This was conducted by labeling the primary antibody, anti-TH mouse monoclonal antibody (1:500, Cat#22941, Immunostar, United States) with biotinylated anti-mouse immunoglobulin in Tris HCl buffer for 20 min followed by the addition of normal mouse serum as the blocking reagent to bind to the residual unbound biotinylated antibody. Upon antibody incubation, the tissue was incubated with streptavidin-peroxidase, followed by a reaction with diaminobenzidine (DAB)/hydrogen peroxidase reaction as the substrate-chromogen as per manufacturer protocol. Stained tissue was mounted with coverslips and viewed under light microscopy. The TH positive (TH+) cells were analyzed based on overall positive cell groups that have been reported previously (Rink and Wullimann, 2002; Wullimann and Rink, 2002; Filippi et al., 2010; Yamamoto et al., 2010). Inter- and intra-rater validation of the microscopic evaluation of the chromogenic staining was conducted by an expert pathologist and an anatomist where the tissue sections were blinded, and positive cells were counted. The results were analyzed to confirm the cell count method used. The regions that were analyzed included the olfactory bulb (OB), subpallidum (SP), pretectum (PR), preoptic region (PO), ventral thalamus (VT), paraventricular organ (PVO), and periventricular nucleus of posterior tubercle (TPp) of the posterior tuberculum (PT).

### 2.7. Statistical analysis

The data received was updated in the Statistical Package for the Social Sciences version 20 software (SPSS Inc., United States). All data were expressed as mean ± standard error of mean (SEM). The data were plotted to evaluate the normality based on a histogram plot, z value, and Shapiro–Wilk test. A suitable statistical analysis was chosen based on the normality plot and the aim of the study. Data for locomotor assessment, gene expression, and protein level. TH+ cell counts were normally distributed. These data were expressed as mean ± SEM and analyzed with One-Way Analysis of Variance (ANOVA) and *post hoc* Tukey’s test. In view of multiple group comparisons, independent-sample *t*-test were used for paired comparisons between two specific groups. A *p* value of <0.05 is considered statistically significant.

## 3. Results

### 3.1. Locomotor assessment

**Figure 2:**
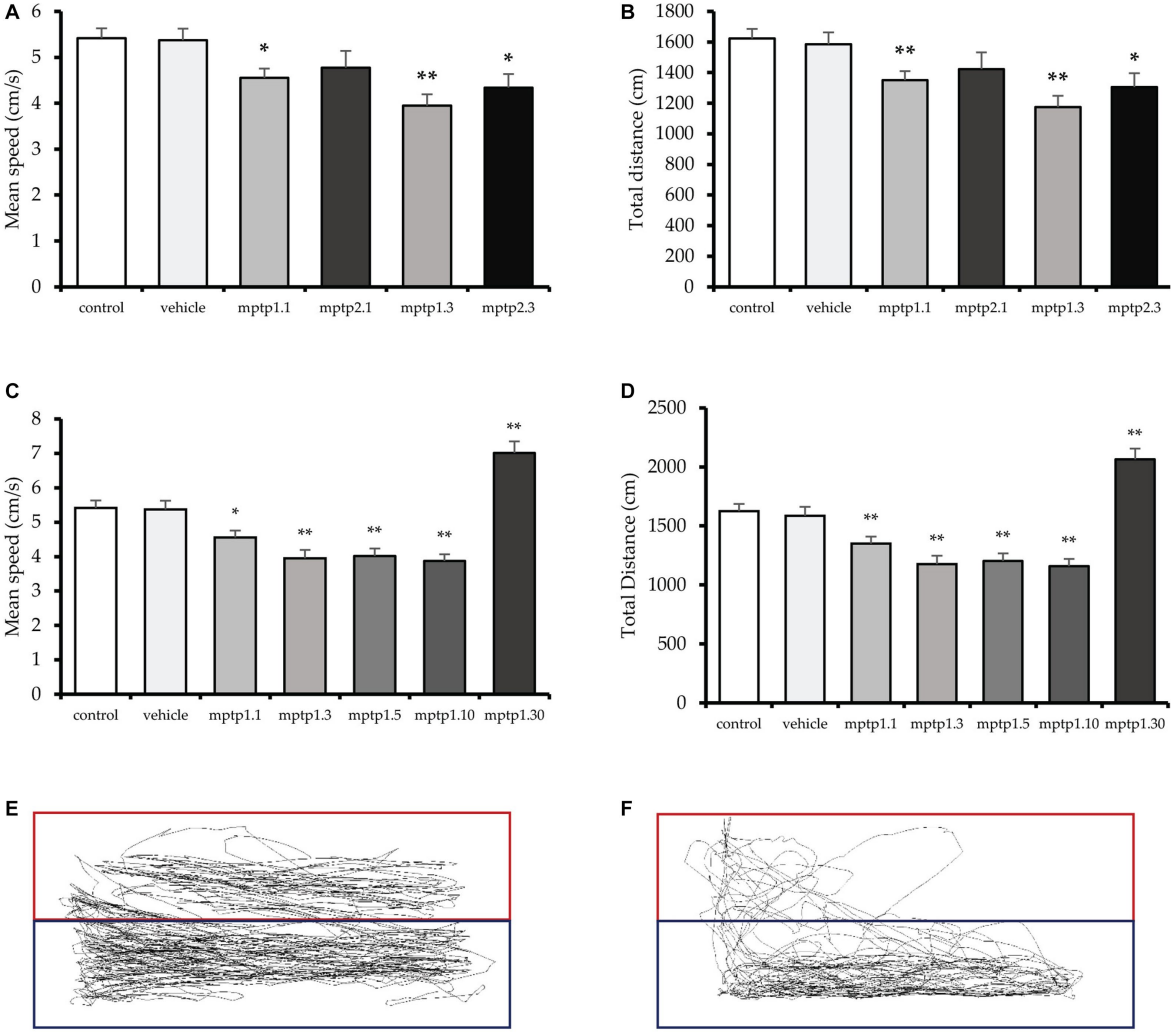
The locomotor assessment of the zebrafish models. **(A,B)** Total distance traveled and speed of the zebrafish between one- and two-times injection groups. **(C,D)** Total distance traveled and speed of one-time injection groups from day 1 to 30 post-MPTP injection. The deepest dip in speed and distance can be appreciated at day 3 post-injection, which is then plateaued until day 10. There is no difference in speed and distance traveled between day 3 to 10 groups. **(E)** Tracking of the swimming pattern in the control. **(F)** Tracking for group MPTP1.3. The exploration of the top tank is more substantial in the control group. Data are presented as mean ± SEM. Asterisks (*) indicate a significant difference between vehicle and treated fish at *p < 0.01 or **p < 0.05.

The mean swimming speed was affected from day 1 post-MPTP injection, where there was a 16 and 12% reduction in MPTP1.1 (4.56 ± 0.20; *p* = 0.026) and MPTP2.1 (4.78 ± 0.36; *p* = 0.151) groups, respectively. However, only one-time injection reached statistical significance. Similarly, there were 27 and 20% reductions in mean speed in day 3 assessment for one- and two-time injection groups, respectively, with MPTP1.3 (3.95 ± 0.24; *p* < 0.001) and MPTP2.3 (4.35 ± 0.40; *p* = 0.015). There was no significant difference in the mean speed between the groups on the same assessment day, but different injection frequencies. Subsequently, the mean speed was monitored for an extended period until day 30 post-injection. There was a 26% reduction in MPTP1.5 (4.02 ± 0.21; *p* < 0.001) and a 29% reduction in MPTP1.10 (3.88 ± 0.19; *p* < 0.001). However, the swimming velocity improved significantly with an increment in speed of 26% from the control in the MPTP1.30 group (7.01 ± 0.31; *p* < 0.001) (Figure 2).

Coherently, the total distance swam followed a similar pattern with the speed. It was observed that total distance was affected from day one post-injection MPTP1.1 (1350.15 ± 58.35; *p* = 0.002), where there was a 17% reduction in distance observed, and 12% reduction in MPTP2.1 (1422.96 ± 109.78; *p* = 0.126), however, only one-time injection group reached statistical significance. The distance traveled was further affected on day 3, with a 28% reduction in the distance in MPTP1.3 (1175.32 ± 72.64; *p* < 0.001) and a 20% reduction in the distance in MPTP2.3 (1305.04 ± 90.78; *p* = 0.015). There was no significant difference in one- and two-times injection group findings on similar assessment days (Figure 2). The swimming distance plateaued from day 3 to day 10, where there was a 26% reduction in the distance in MPTP1.5 (1203.10 ± 64.39; *p* < 0.001) and a 29% reduction in MPTP1.10 (1160.30 ± 59.00; *p* < 0.001). There was no statistical significance difference among these three groups. On day 30, there was a significant boost in swimming distance, with a 27% increase in distance compared to control in the MPTP1.30 group (2064.57 ± 38.51; *p* = 0.001) (Figure 2).

It was observed that the mean speed and total distance traveled from day 1 to day 30 were significantly different statistically (*p* < 0.001). We have also assessed the swimming pattern to observe the explorative capabilities of the fish across the groups. Although there were some fluctuations in the readings for the percentage of time spent on the top tank and the latency to reach the top, the results were not statistically significant. This could be due to the high variability of the findings, which has affected the SEM reading. The findings of all locomotor parameters between the control and vehicle groups were statistically not significant (Supplementary Figure S1).

### 3.2. Gene expression

**Figure 3:**
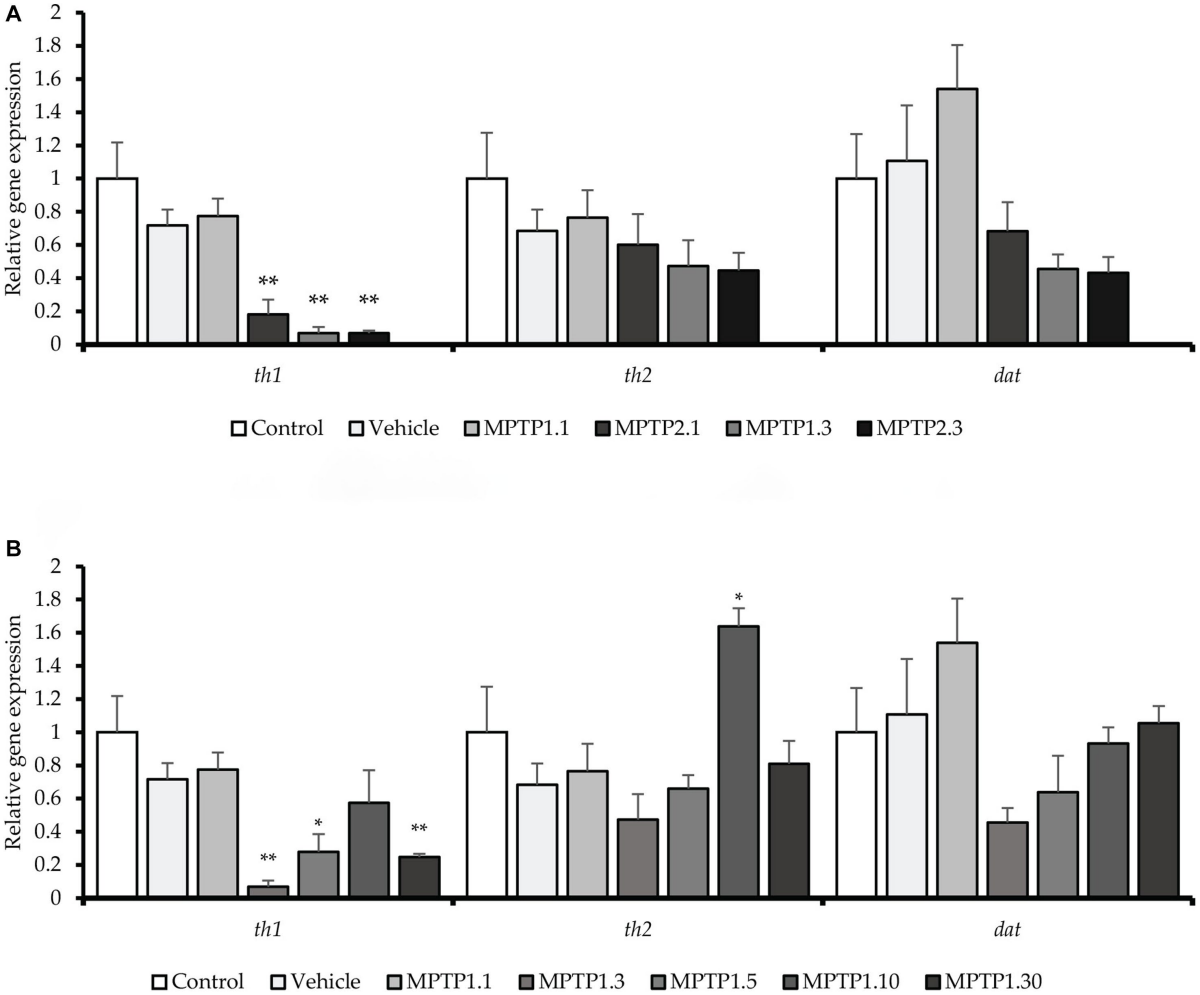
The relative gene expression in the zebrafish model across the group. *th1* gene was significantly down-regulated in MPTP1.3, MPTP1.5, MPTP2.1, and MPTP2.3. *th2* gene expression was down-regulated initially however was not statistically significant. However, at MPTP1.10, there is a spike in *th2* gene expression which eventually down-regulated in MPTP1.30. *dat* gene was down-regulated, especially in MPTP1.3 and MPTP2.3 then steadily increased afterward; however, these changes were not statistically significant. **(A)** The relative gene expression between one- and two-time injections. **(B)** The relative gene expression among MPTP days 1 to 30. Data are presented as mean ± SEM. Asterisks (*) indicate a significant difference between vehicle and treated fish at *p < 0.01 or **p < 0.05.

Gene expressions were measured relatively using quantitative real-time PCR against the *actb1* gene (Rassier et al., 2020). All gene markers that were tested showed no statistical difference between the control and the vehicle group. There has been significant dysregulation of the gene, especially the *th1* gene across the treatment group. In contrast to the locomotor findings, the gene dysregulation was not evident at day 1 post-injection for one-time MPTP injection, MPTP1.1 (0.78 ± 0.10; *p* = 0.30), however, it was significantly down-regulated in the two-times injection group, MPTP2.1 (0.18 ± 0.08; *p* = 0.006). On day three of the assessment, both one- and two-time injection groups showed down-regulation of the *th1* gene, MPTP1.3 (0.07 ± 0.03; *p* = 0.003) and MPTP2.3 (0.07 ± 0.01; *p* = 0.001) (Figure 3). The differences between one- and two-times injections on day 3 of the assessment were not statistically significant. *th1* gene expression was observed to incline afterward at day 5, albeit still down-regulated from the vehicle group, MPTP1.5 (0.28 ± 0.10; *p* = 0.029). This continued on day 10, where the differences in expression were not statistically significant compared to the vehicle group, MPTP1.10 (0.57 ± 0.20; *p* = 0.276). On day 30 of the assessment, the *th1* gene was significantly down-regulated compared to the vehicle group (0.25 ± 0.02; *p* = 0.007). Comparing *th1* gene expression over the period of 30 days, the differences were significant (*p* < 0.001) where there was a deepest dip in the *th1* expression during day 3 to 5, then slowly improving, however, to be down-regulated again at day 30. We could observe similar trends in *th2* and *dat* gene expression (Figure 3). However, none of the groups reached a statistically significant difference from the control or vehicle group and within the same injection frequency or assessment day, except for MPTP1.10 where the *th2* gene was significantly up-regulated (1.64 ± 0.11; *p* = 0.021) (Table 3).

**Table 3:** Mean relative gene expression in zebrafish whole brain.

Gene	Control	Vehicle	MPTP 1.1	MPTP 2.1	MPTP 1.3	MPTP 2.3	MPTP 1.5	MPTP 1.10	MPTP 1.30
*th1*	1.00 ± 0.22	0.72 ± 0.10	0.78 ± 0.10	0.18 ± 0.08^*^	0.07 ± 0.03^**^	0.07 ± 0.01^**^	0.28 ± 0.10^*^	0.57 ± 0.20	0.25 ± 0.02^**^
*th2*	1.00 ± 0.28	0.68 ± 0.12	0.77 ± 0.17	0.60 ± 0.18	0.47 ± 0.15	0.45 ± 0.11	0.66 ± 0.08	1.64 ± 0.11^**^	0.81 ± 0.14
*dat*	1.00 ± 0.27	1.10 ± 0.33	1.54 ± 0.27	0.68 ± 0.17	0.46 ± 0.09	0.43 ± 0.09	0.64 ± 0.22	0.93 ± 0.09	1.06 ± 0.10

*actb1* gene was used as the reference gene. Data are presented as mean ± SEM. Asterisks (*) indicate a significant difference between control and treated fish at *p < 0.05 or **p < 0.01.

### 3.3. Protein level

**Figure 4:**
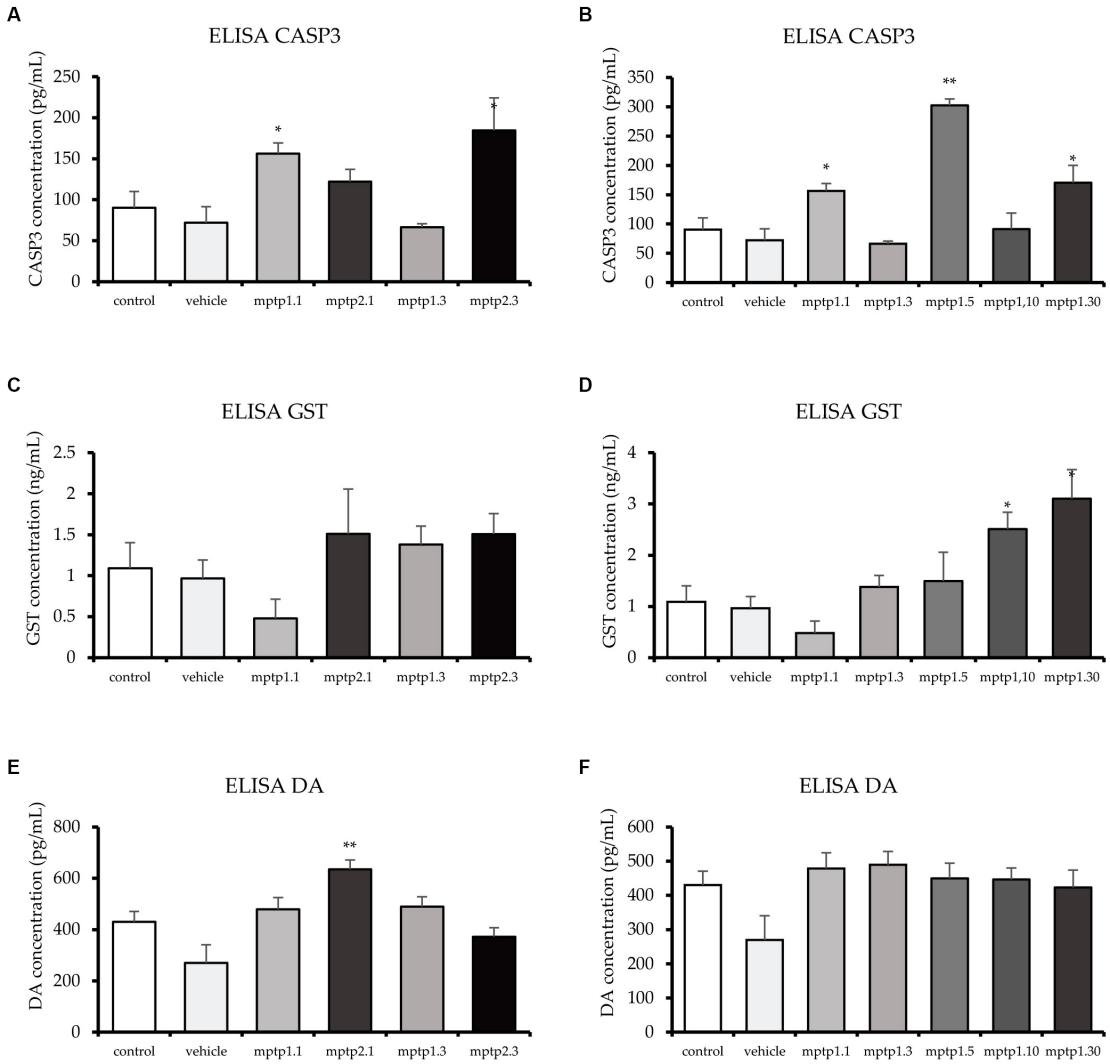
ELISA test for CASP3, GST and DA. CASP3 levels fluctuated across the group, with MPTP1.1 and MPTP1.5 significantly elevated. The GST protein was assessed and showed a sudden drop in MPTP1.1, albeit statistically insignificant. Henceforth, the level significantly increased in the day 10 and day 30 groups. DA levels were equivocal except for MPTP2.1 where it is significantly elevated. **(A,C,E)** ELISA results between one- and two-time injection. **(B,D,F)** Result between days of assessment from day 1 to 30. Data are presented as mean ± SEM. Asterisks (*) indicate a significant difference between vehicle and treated fish at *p < 0.01 or **p < 0.05.

Whole zebrafish brains were analyzed for four proteins, namely DA, CASP3, GST, and BDNF. CASP3 and GST were assessed in view of their vital roles in apoptosis and cellular protection in oxidative stress (Saleem et al., 2019; Silva et al., 2020; Omoruyi et al., 2021). CASP3 has been shown to significantly double its level in the MPTP1.1 and MPTP2.3 groups. MPTP2.1 (122.19 ± 14.81) has shown elevated CASP3, almost similar to the former two groups, but did not reach statistical significance. Interestingly, MPTP1.3 has a slightly lower CASP3 level compared to the control and vehicle groups, however, it was not statistically significant. Comparisons between one- and two-time injections were only significant at day 3 assessment with a *p*-value of 0.041 (Table 4). Subsequently, MPTP1.5 (302.40 ± 11.00) had a remarkable increment in CASP3 level of 4.2 times higher than the vehicle group significantly. This was then dropped again to the control level on day 10, only to double again on day 30 (Figure 4). These rather extreme changes over 30 days have been shown to be statistically significant with a *p*-value of <0.001.

**Table 4:** Protein level from ELISA test.

Protein	Control	Vehicle	MPTP1.1	MPTP2.1	MPTP1.3	MPTP2.3	MPTP1.5	MPTP1.10	MPTP1.30
DA (pg/mL)	430.42 ± 40.60	279.88 ± 71.08	478.89 ± 45.53	635.52 ± 36.27^**^	489.61 ± 38.72^*^	372.23 ± 34.98	449.99 ± 44.18	446.74 ± 33.56	423.62 ± 50.14
CASP3 (pg/mL)	90.30 ± 19.88	72.01 ± 19.64	156.34 ± 13.01^*^	122.19 ± 14.81	66.47 ± 4.34	184.61 ± 39.61^*^	302.40 ± 11.00^**^	91.21 ± 27.19	170.64 ± 29.45^*^
GST (ng/mL)	1.09 ± 0.96	0.966 ± 0.23	0.96 ± 0.20	1.51 ± 0.54	1.38 ± 0.23	1.51 ± 0.25	1.99 ± 0.37	2.51 ± 0.33^*^	3.11 ± 0.56^*^
BNDF (pg/mL)	331.49 ± 23.64	369.654 ± 19.08	350.52 ± 14.17	301.56 ± 25.35	320.82 ± 35.85	319.56 ± 28.05	348.82 ± 44.51	335.27 ± 12.58	339.49 ± 26.43

Data are presented as mean ± SEM. Asterisks (*) indicate a significant difference between vehicle and treated fish at *p < 0.05 or **p < 0.01.

In contrast to CASP3, the GST level has steadily risen following MPTP injection. One- and two-time injection groups did not reach statistically significant differences when compared with either vehicle group or within similar assessment date groups. Following that, the day 5 group almost doubled its GST level (1.99 ± 0.37) but did not reach statistical significance. It was not until day 10 that the differences in GST levels reached statistically significant results, where MPTP1.10 had 2.5 times and MPTP1.30 had 3.2 times higher GST levels compared to the vehicle group. The steady rise of GST levels over the period of 30 days has been shown to be statistically significant with a *p*-value of 0.002 (Figure 4).

DA levels of all groups were found to be equivocal except for MPTP2.1 (635.52 ± 36.27; *p* = 0.002) and MPTP1.3 (489.61 ± 38.72; *p* = 0.043) where the DA level was peculiarly increased by triple and double respectively, compared to the vehicle group level. The comparison of this group to the one-time injection group, MPTP1.1, was significantly different, with a *p*-value of 0.022. The extended observation days had equivocal results for the control and vehicle groups (Figure 4). Comparison of one-time injection over the period of 30 days was statistically insignificant in their DA level (*p* = 0.225). BDNF was assessed as a protective neurotrophic factor that is involved in neuronal regeneration and repair. In this study, the BDNF level showed an almost similar pattern to the DA level, where all groups had equivocal levels. Comparisons between one- and two-time injections and comparisons between days 1 to 30 were all statistically insignificant (Table 4; Supplementary Figure S2).

### 3.4. Immunohistochemistry assessment

**Figure 5:**
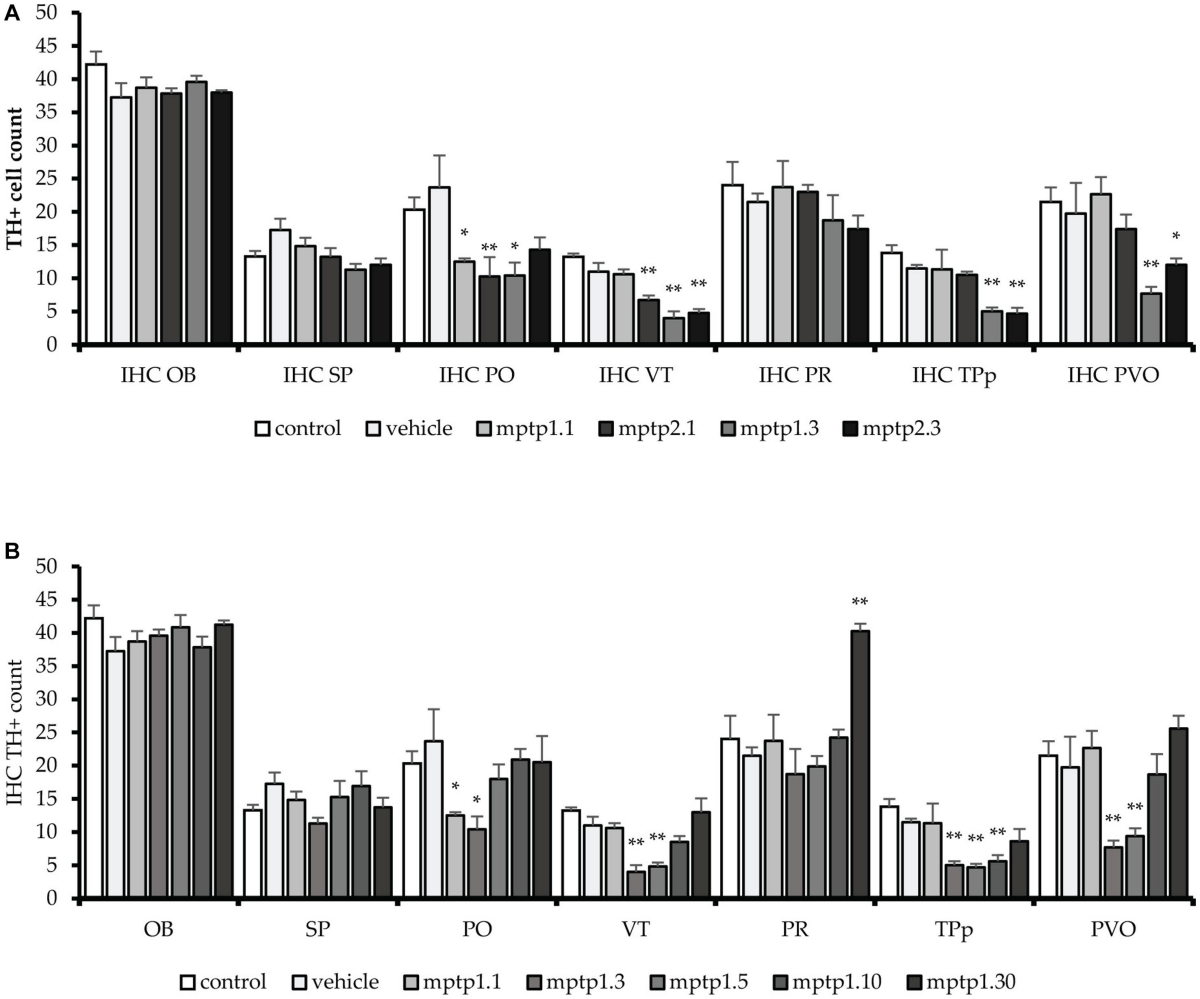
TH+ cells count in different regions of the brain. MPTP in this study has been shown to affect a few areas of dopaminergic neurons, sparing the OB, SP and PR areas. VT and PO area has been seen to be affected the earliest, as early as day 1. Subsequently, other areas were affected. The pattern is similar to other markers tested before, where the lowest counts were noted in MPTP1.3 and MPTP1.5. Day 10 assessment has shown recovery of the cell counts, which mostly return to the same counts as in control on day 10. Although cell counts did not reduce in pretectum following MPTP insult, it is noted that on day 30, the PR cell counts were significantly increased. **(A)** TH+ cell between one- and two-times injection. **(B)** TH+ cells between days of assessment from day 1 to 30. Data are presented as mean ± SEM. Asterisks (*) indicate a significant difference between vehicle and treated fish at *p < 0.01 or **p < 0.05.

**Figure 6:**
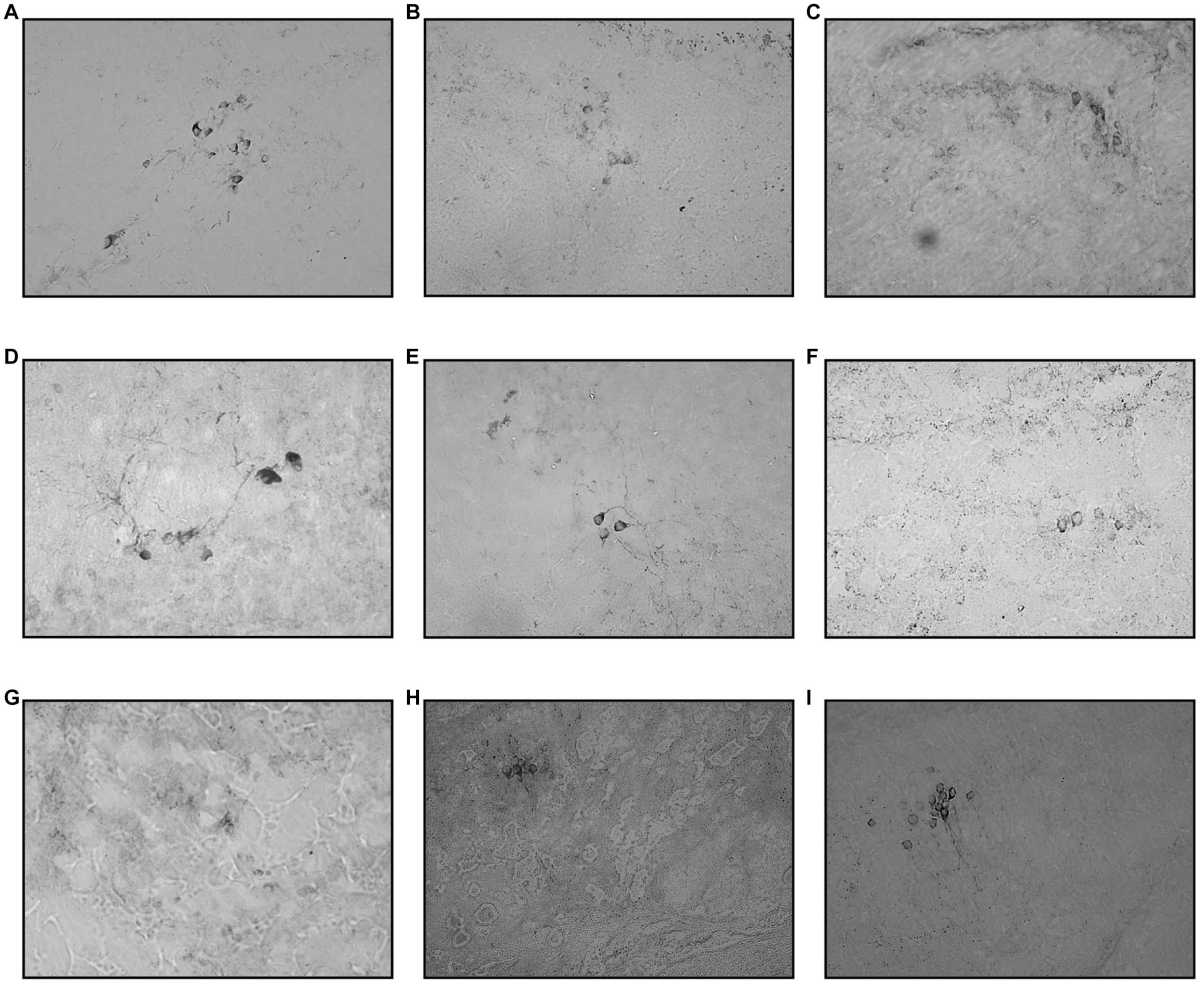
Coronal section of ventral thalamus. The TH+ cells were marked by a deep chromogenic stain. The cell number can be seen declining from day one and peaked around days 3 and 5. Day 10 and Day 30 sections showed re-emerging of positive TH+ cell stain. **(A)** Control, **(B)** Vehicle, **(C)** MPTP1.1, **(D)** MPTP2.1, **(E)** MPTP1.3, **(F)** MPTP2.3, **(G)** MPTP1.5, **(H)** MPTP1.10, **(I)** MPTP 1.30.

The immunohistochemistry assessment using chromogenic stain against TH protein was based on TH+ cell counts in all regions known to express dopaminergic neurons, as previously described. There was no statistical difference in TH+ cell counts between the control and vehicle group tissue sections. VT has been shown to be one of the earliest regions to be affected by MPTP injection, where there is a drop of 11% and a significant drop of 41% of TH+ cells 1 day following one- and two-time injections of MPTP, respectively. The differences in TH+ cell counts on day one of assessment in these two groups were statistically significant. Which further dropped 63% from control during the day 3 assessment for both one- and two-time injection groups. Following that, the day 5 assessment showed a slight improvement in TH+ cell counts, where there was a 56% reduction of TH+ cells compared to the vehicle group. The recovery is more evident on day 10, where only 22% of TH+ dropped compared to the vehicle group. By day 30, the TH+ cell count had resumed, similar to the control, and the counts were not statistically significant (Figures 5, 6).

Similarly, the PO region has shown a similar pattern to the VT cell count loss. There was a significant reduction of 45% and 69% in TH+ cell counts following 1 day of injection in the one- and two-time injection groups, respectively. Again, like in VT, the differences between these two groups were statistically significant. Day 3 assessment in one- and two-time injection showed a slight difference, where only one-time injection showed a significant drop of 56% of positive cell counts, while two-time injection cell counts only dropped 40%. However, the differences between these two groups did not reach statistical significance. Following that, the recovery in this region has occurred swiftly compared to VT, where there was only a 23% reduction in positive cell counts in the MPTP 1.5 group compared to the vehicle group. Subsequently, the day 10 and 30 groups have shown to resume the TH+ cells similar to the control and vehicle groups.

**Figure 7:**
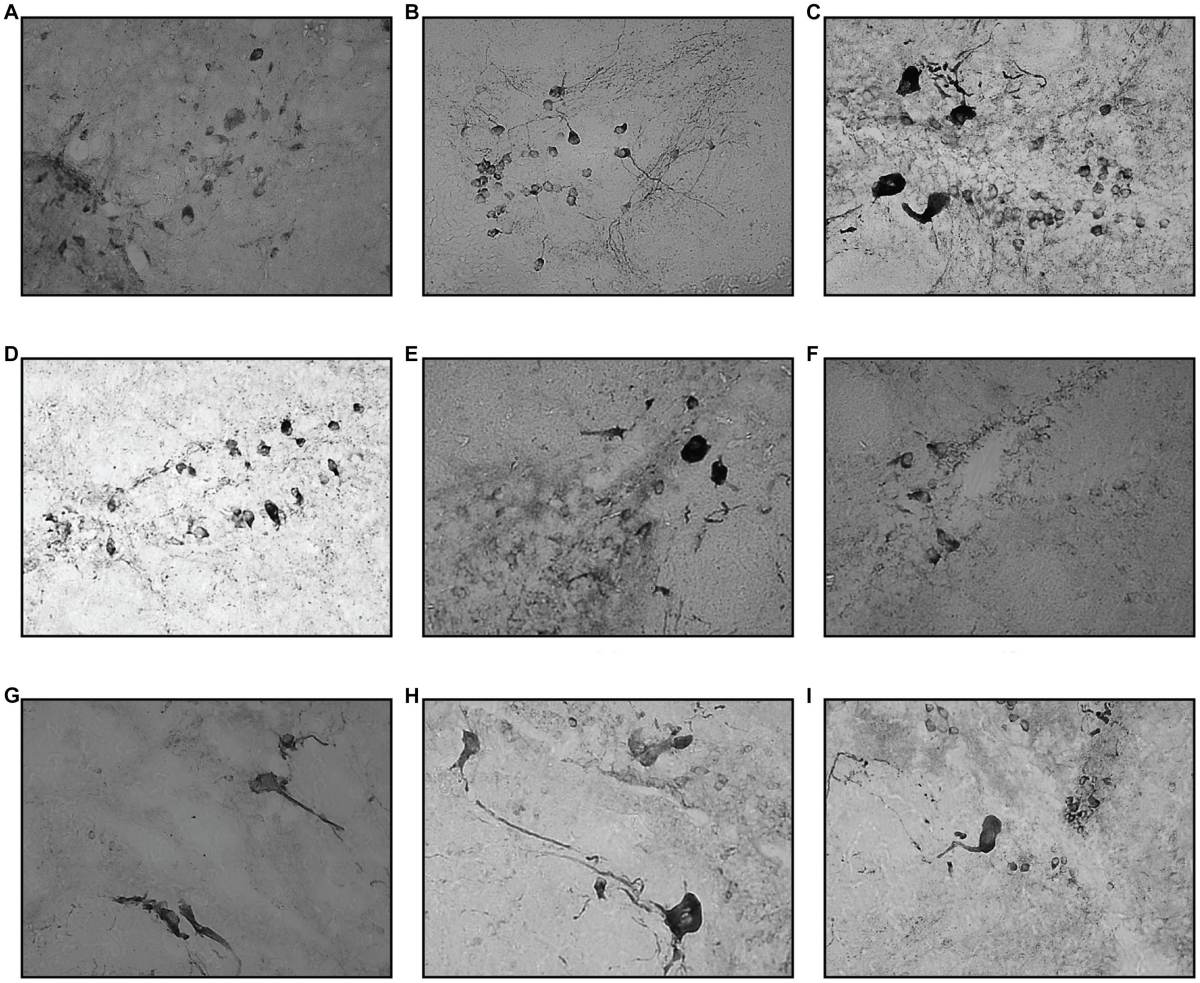
Coronal section of TPp and PVO. Compared to VT, the drop in TH+ cell counts were more evident starting from day 3, for both one- and two-time MPTP injections. This finding persisted on day 5 of the assessment. Day 10 tissue section showed a slight improvement in TH+ cell count, which progressed eventually during the day 30 assessment. **(A)** Control, **(B)** Vehicle, **(C)** MPTP1.1, **(D)** MPTP2.1, **(E)** MPTP1.3, **(F)** MPTP2.3, **(G)** MPTP1.5; **(H)** MPTP1.10, **(I)** MPTP 1.30.

In the PT region, TPp and PVO were assessed. In the TPp region, during day one of the assessment, although both groups showed a reduction in TH+ cell count, neither reached statistical significance when compared with the vehicle group. In addition, there was no significant difference in the cell counts between one- and two-time injections on day one of assessment. TH+ cell counts were drastically reduced by 60% compared to the vehicle group in the day 3 assessment for both one- and two-time injection groups, which plateaued during days 5 and 10 of the assessment. In contrast to VT and PO, day 30 of the assessment has not resumed TH+ cell count, where there is still a significant drop in TH+ cell counts by 25% compared to the vehicle group. Similarly, in PVO, during the day one assessment, only MPTP 2.1 had a significant reduction in TH+ cell count by as much as 10%. However, there is no significant difference between one- and two-time injections on day 1 assessment. Following that, there is a drastic drop in TH+ cell count at day 3 assessment in both one- and two-time injections, 61 and 40%, respectively. From thereon, the TH+ cell count steadily rose, with a significant 9% improvement in TH+ in MPTP 1.5 compared to MPTP 1.3. From day 10 onwards, the TH+ cell count continues to rise, and there is no significant difference in the TH+ cell count in MPTP 1.10 and MPTP 1.30 compared to the control and vehicle groups (Figures 5, 7).

The PR region has shown similar TH+ cell counts in all groups except MPTP 1.30, with a significant increment in the doubling of TH+ cell counts compared to the vehicle group. OB and SP have been shown to have equivocal TH+ cell counts in all MPTP groups, and there is no significant difference when comparing one- and two-time injections or between day one to day 30 of the assessment. Assessment of the groups from day one to 30 was significantly different selectively at VT, TPp, PVO, and PR (Table 5; Supplementary Figure S3).

**Table 5:** TH+ cell count in different regions of the zebrafish brain.

Region	Control	Vehicle	MPTP1.1	MPTP2.1	MPTP1.3	MPTP2.3	MPTP1.5	MPTP1.10	MPTP1.30
OB	42.20 ± 1.93	37.25 ± 2.12	38.71 ± 1.56	37.84 ± 0.74	39.60 ± 0.92	38.00 ± 0.32	40.87 ± 1.80	37.82 ± 1.61	41.25 ± 0.63
SP	13.30 ± 0.82	17.25 ± 1.70	14.81 ± 1.26	13.22 ± 1.31	11.29 ± 0.86	12.00 ± 0.98	15.27 ± 2.41	16.91 ± 2.23	13.71 ± 1.46
PO	20.33 ± 1.86	23. 67 ± 4.84	12.50 ± 0.50^*^	7.33 ± 0.33^**^	10.42 ± 1.92^*^	14.29 ± 1.86	18.00 ± 2.16	20.89 ± 1.62	20.50 ± 3.96
VT	13.25 ± 0.47	12 ± 1.30	10.60 ± 0.74	6.71 ± 0.68^*^	4.00 ± 1.00^**^	4.75 ± 0.63^**^	4.80 ± 0.58^**^	8.50 ± 0.87	13.00 ± 2.08
PR	24.00 ± 3.53	21.5 ± 1.25	23.75 ± 3.90	23.00 ± 1.08	18.71 ± 3.82	17.40 ± 2.09	19.90 ± 1.56	24.20 ± 1.24	40.25 ± 1.10^**^
TPp	13.80 ± 1.20	11.5 ± 0.5	11.33 ± 1.20	10.50 ± 0.50	5.00 ± 0.58^**^	4.67 ± 0.89^**^	4.67 ± 0.56^**^	5.60 ± 0.91^**^	8.60 ± 1.86^*^
PVO	21.50 ± 2.18	19.75 ± 4.58	22.67 ± 2.56	17.40 ± 2.18	7.67 ± 1.04^**^	12.00 ± 1.00^*^	9.40 ± 1.16^**^	18.67 ± 3.07	25.60 ± 1.94

Data are presented as mean ± SEM. Asterisks (*) indicate a significant difference between vehicle and treated fish at *p < 0.05 or **p < 0.01.

## 4. Discussion

### 4.1. Frequency of injection and duration of induction

MPTP injection in animal models has been shown to mimic the PD phenotype. Our initial concern was to optimize the MPTP protocol specifically for a zebrafish animal model. This is because, despite having many benefits in terms of high fecundability and similarities with the human genome, its ability to regenerate its central nervous system makes it a face-to-face battle between choosing the right time of assessment following MPTP induction, as reported by (Sarath Babu et al., 2016) in their paper, where they induced PD in zebrafish with MPTP with either one- or two-time injections and were assessed 24 h later. Their study has shown that two-time injected zebrafish have significant PD features on more positive markers than one-time injection. Based on this study, we have conducted the first part of our study. However, we reported a similar result between the one-time and two-time injection groups. We then extended the study to assess the zebrafish for another 2 days. This is when we saw significant changes, however, not between one- and two-time injections, but between the days of assessment, where day 3 post-MPTP injection has shown statistically significantly reduced swimming speed, a total distance of swimming, reduced *th1* and *dat* gene expression and reduced TH+ cells in immunohistochemistry, regardless of frequency of injection.

We then extended the assessment for up to 30 days to review the benefits of delaying the assessment. Hence, in the second part of the study, we analyzed the fish on days 5, 10, and 30 post-MPTP injections. From this assessment, it has been shown coherently for all markers, that the day 3 to day 5 group has shown the most statistically significant changes in locomotion, *th1* and *dat* gene expression, and TH+ neuronal degenerated cells. From that moment on, improvement in these parameters have been seen from day 10 onwards, and by day 30, the parameters are comparable to the control group. In the day 30 assessment, locomotor activity was higher than in the vehicle group, with significant recovery in TH+ cell counts, especially in the VT, PO, and PT regions. The *th1* gene was seen to be down-regulated compared to the vehicle group, but looking at the pattern, this happened following a peak in *th1* expression during day 10. The recovery features were more evident with the steady inclining of the GST level. Although the caspase-3 level was significantly higher than the vehicle group, looking at the fluctuating pattern of caspase-3, the increase is less pronounced following a dip on day 10. This is similar to a recent study that induced PD in an adult zebrafish group using CVMI injection using 6-OHDA, where they could see the recovery process of the parameter that they tested in the second week following neurotoxin insult (Md Hamzah et al., 2021). In other words, the timing of PD model induction may be more related to the pharmacodynamics of the drugs in the zebrafish model than to the drug itself. This is particularly important as any form of PD treatment to be given post-MPTP induction should not exceed day 10, when the fish can repair and regenerate neuronal cells.

### 4.2. Swimming pattern, bradykinesia, and anxiety

We have analyzed the locomotor assessment based on the total distance of swimming within 5 min and the speed of the swim. These are pathognomonic features of PD where the clinical features of bradykinesia have been reflected in the swimming speed and distance. In this study, it has been shown that regardless of the day and frequency of injection, MPTP has successfully reflected the typical neurobehavioral feature in the PD zebrafish model, which is bradykinesia (reduction in mean speed of swimming). There is a slight increment in mean speed and total swimming distance in the two-time injection group when compared to the one-time injection group. Although the differences did not reach statistical significance, it is rather peculiar to see an increment rather than a further drop in the two-time injection group. This could be the effect of multiple anesthesia that could induce stress or anxiety in this group. A study conducted on the effects of anesthesia on the serum cortisol level of teleost fish has found that the time of anesthesia corresponds 96% with the increment of the serum cortisol level (Wosnick et al., 2018). This in turn could be presented as an acute stress response in the zebrafish group. A review conducted looking at the fish response to acute and chronic stress has summarized that acute stress in fish could be presented as a higher swimming distance compared to control (Demin et al., 2021). However, we did not further explore the analysis as this was beyond the scope of the current study.

Apart from that, we have also analyzed the latency of the fish to reach the top half of the tank and the percentage of time spent in the top half of the tank. These are markers to show the explorative abilities of the fish where anxiety could lead to less explorative capability of the fish, hence less time on the top half of the tank and longer time needed for them to explore the top half (latency to reach the top) (Robinson et al., 2013). In our study, MPTP1.3 to MPTP1.10 have shown some reduction in the percentage of time spent by the fish on the top half of the tank, and coherently, it takes a longer time for these groups for the first to start and explore the top half for the first time during the assessment. This is in accordance with a few studies showing non-motor PD behaviors in adult zebrafish following induction with neurotoxins (Wang et al., 2017; Selvaraj et al., 2019). However, the results were not statistically significant due to some variation in results within the same group that resulted in a higher SEM value. PD is associated with numerous non-motor symptoms, i.e., mental illness, cognitive dysfunction, and pain (Samat et al., 2017; Carapellotti et al., 2020). The anxiety symptoms in PD patients have also been well reported, with anxiety reported in 25.7% of PD patients (Broen et al., 2016b). The fact that the pattern of anxiety features in zebrafish and PD-specific features is similar may indicate the possibility that the neurochemical pathway plays a bigger role in inducing anxiety in PD patients than previously thought (Broen et al., 2016a).

### 4.3. Gene expression in the PD model

As reported by Yamamoto et al., as a result of teleost whole genome duplication, there have been two *th* genes found in zebrafish species. It has been found that *th1* gene were more abundant in the brain and eye regions, while *th2* were more abundant in the liver, gills, heart and kidney of the zebrafish, with *th2-*expressing cells in the brains being more confined in the diencephalic region (Chen et al., 2009; Filippi et al., 2010). In the diencephalon, neurons expressing both these genes are dopaminergic neurons that were located either co-existing, like in the PT and PO, or in separate areas (Yamamoto et al., 2010). Whilst *dat* gene is responsible for the synthesis of DAT protein, which is vital for DA metabolism, uptake and release in the neuron (Vaughan and Foster, 2013). Our findings of selective *th1* and *dat* gene down-regulation sparing the *th2* gene were similar to a previous study that induced PD in a zebrafish model using paraquat (Mohamad Najib et al., 2021). It is also important to note that, in PD, only certain regions of dopaminergic neurons are affected, hence these locations are essential to understanding why only certain *th1* genes are affected by the neurotoxin. It was mentioned in previous studies that the location of *th2* cells is more abundant in the caudal hypothalamic region (Filippi et al., 2010; Yamamoto et al., 2010; Semenova et al., 2014), which is not directly related to the regions affected in the zebrafish PD model. It could also be due to the slightly different function of *th1* compared to *th2*-expressing neurons. Studies have linked *th2* with stress-related studies, which showed the up-regulation of *th2* genes and staining in response to stress in zebrafish (Pavlidis et al., 2011; Semenova et al., 2014).

Looking at the pattern of gene expression throughout the 30 days post-injection period, the gene expression follows a similar pattern whereby the least down-regulation was seen on day 3, and following that, gene expression has been seen to increase the most by day 10. Following that, the gene expression has either returned to similar levels to control or been slightly down-regulated. On day 10, the *th2* gene expression was markedly up-regulated and was the only day where *th2* expression was significantly different from the control and vehicle groups. Similarly, the *th1* gene was up-regulated the most on a similar day following MPTP injection. This could be part of the neuroregeneration effort by the zebrafish to recover from the neurotoxic effects of the MPTP. This is consistent with a study conducted by Yamamoto et al., where they established that both TH1 and TH2-containing cells are responsible for synthesizing DA. It is also worth noting that, albeit not abundantly, there are regions in the brain, especially in the paraventricular region, where TH1- and TH2-expressing cells coexist (Filippi et al., 2010; Yamamoto et al., 2010).

### 4.4. Protein expression in the PD model

We also conducted ELISA assessments measuring the level of protein in the zebrafish brain for all the groups. The first protein examined is DA, which is one of the catecholamine neurotransmitters, aside from noradrenergic and adrenergic (Bhat and Ganesh, 2017). DA is the activated protein processed from tyrosine by the enzyme TH. It has been shown that DA levels change in conditions like PD and anxiety in zebrafish. In our study, DA levels were measured from the whole zebrafish brain, which has shown no significant difference compared to the control group. There could be a few reasons for this, for instance, it is worth noting that dopaminergic neurons have a specific function in a specific region of the brain. Hence, the result may vary depending on whether the assessment is done as a whole brain or in a specific brain region. Another reason could be that the anxiety features that have been shown in locomotor assessment could be reflected as elevated DA levels (Kacprzak et al., 2017; Liu et al., 2020) in the hypothalamus of the zebrafish brain (Filippi et al., 2010; Yamamoto et al., 2010; Semenova et al., 2014) (which were not measured in this study), that have been reflected as equivocal DA level during the assessment of the whole brain. MPTP used in the study has also been known for its selective degenerative effects in PD dopaminergic clusters, which may not reflect on the total DA level in the whole zebrafish brain (Pollard et al., 1996; Storch et al., 2004; Langston, 2017).

CASP3 is one of the cysteine proteases that play an essential role in the apoptotic signaling pathway and is known as the primary executioner of cell death (Valencia et al., 2007; Brentnall et al., 2013). It has been found that CASP3 is responsible for the proapoptotic cascade in both intrinsic and extrinsic pathways (Pyati et al., 2007). Studies have proven the role of CASP3-induced cell apoptosis following MPTP injection in their *in vivo* studies (Turmel et al., 2001; Yamada et al., 2010; Spead et al., 2018). In our study, the CASP3 level from ELISA peaked significantly on day 1 post-injection and subsequently fluctuated as the day progressed. Towards day 30, the level remained higher than in the vehicle group. The increment of CASP3 following MPTP injection had a similar result in a recent study, which showed a significant increment of CASP3 in their colorimetric or western blotting assessment of the protein (Liang et al., 2019; Haque et al., 2021). The last protein that was assessed was GST. This intracellular thiol compound can be found in the cytosol and mitochondria and plays a crucial role in defense against respiration-induced reactive oxygen species (ROS) (Ribas et al., 2014). The vital redox agent for ROS is generated mainly in complexes 1 and 3 of the electron transport chain (Ribas et al., 2014). The GST level showed a sudden drop in the day 1 assessment, which followed the pattern of the peak in CASP3 on the same day, however, this level did not reach statistical significance. Following that, there is a steady rise in GST level, significantly from day 10 onwards. The steady increment in GST with fluctuating CASP3 levels could be a sign of non-apoptotic CASP3 activity that could be activated following the regenerative and healing process in the zebrafish brain (Imbriani et al., 2019; Eskandari and Eaves, 2022).

Immunohistochemistry assessments of the zebrafish brain were conducted using chromogenic TH stain. It is vital to note that TH enzymes are located in the cytosol and cell membrane of the cells. Hence, negative TH+ could be a sign of cell degeneration in the regions that are expected to be positive. Among all the regions assessed, the areas affected were specific to VT, PO, TPp, and PVO, while sparing other regions, namely OB, SP, and PR. Locus coeruleus was not analyzed in this study as this TH+ region is the regional expression of noradrenergic catecholamines, which is beyond the scope of the study. The results were coherent with the previous study which has shown that neurotoxin agent causes dopaminergic neurodegeneration specifically in the VT, PO, PVO, and PT while sparing other areas (Caldwell et al., 2019). PT (TPp and PVO) has been reported to represent the dopaminergic neuron in the substantia nigra pars compacta (Matsui, 2017a,b; Matsui and Sugie, 2017; Matsui and Takahashi, 2018). Brown et al. (2021) have also reported the vulnerability of dopaminergic neurons in this region, which is age dependent and was significantly affected following the PINK1 gene knockout-PD model. The VT is one of the first regions to be affected even as early as day one of this study. The pattern of TH+ cell reduction is similar to the previously described parameters, where day 3 has been shown the most marked reduction compared to other groups. The day 30 group has been shown to have a TH+ cell count similar to the control group in all the regions previously affected on earlier days. The peculiar increase in TH+ cell counts in the PR region on day 30 of assessment, where there was a doubling of TH+ cell counts compared to the control or vehicle groups intrigued us for further exploration. The increment could be due to part of the regenerative capacity of the zebrafish itself, where the PR region is one of the regenerative loci that could regenerate TH+ cells that will further migrate centrifugally to the desired locus to replace the degenerated neurons (Grandel et al., 2006). However, we are still unclear on why the other unaffected regenerative loci did not have the same effect, such as in the OB and SP. This could be due to the non-retinorecipient connection of PR to other regions such as the cerebellum and hypothalamus that may have effects on motor function in the zebrafish in order to compensate for the neuronal loss in the PT region which was lower than the control group on the same assessment day (Yanez et al., 2018; Barrios et al., 2020). This is crucial information that needs to be addressed before planning methodologies for treatment research in the PD model.

Most studies have been carried out using MPTP to induce PD in the larval stage of zebrafish, which exhibits reduced dopaminergic neurons and locomotor activity (Najib et al., 2020). Additionally, the larval model is preferred probably because of its small size, which enables high-throughput screening; its transparent body, which is preferred for imaging; similar inflammatory response as in adults; and the exponential phenotypic features induced by small brain alteration (Sallinen et al., 2009; Zanandrea et al., 2020). In this study, we exposed MPTP to adult zebrafish, considering the brain’s ability for regeneration has peaked and the effects of neurotoxins may be observed more closely corresponding to humans. This allows adult zebrafish to provide a more accurate comparison with mammals than their larval counterparts. In contrast, pro-regenerative signals and cell types are known to exist in zebrafish larvae between the larval and adult phases (Alper and Dorsky, 2022).

## 5. Conclusion

Intraperitoneal injection of MPTP has successfully induced PD in an adult zebrafish model. One-time injection with delayed assessment on days 3 to 5 is sufficient to show significant PD features in the zebrafish. The regeneration time shown from day 10 onwards is important to address, especially for studies involving treatment trials for neurodegenerative diseases. Nevertheless, inducing neuroregeneration may serve as a potential therapeutic strategy to treat PD.

## Data availability statement

The original contributions presented in the study are included in the article/Supplementary materials, further inquiries can be directed to the corresponding author.

## Ethics statement

The animal study was approved by Universiti Kebangsaan Malaysia Animal Ethics Committee. The study was conducted in accordance with the local legislation and institutional requirements.

## Author contributions

JK and ST: conceptualization and supervision. ST and NO: methodology. NO, JK, and ST: validation and writing—review and editing. NO: formal analysis, data curation, and writing—original draft preparation. ST: funding acquisition. All authors contributed to the article and approved the submitted version.

## Funding

This research was funded by the Universiti Kebangsaan Malaysia through the Faculty of Medicine Fundamental Grant (grant number FF-2021-177) and the Ministry of Higher Education (MOHE), Malaysia through the Fundamental Research Grant Scheme (grant number FRGS/1/2018/SKK08/UKM/03/5).

## Conflict of interest

The authors declare that the research was conducted in the absence of any commercial or financial relationships that could be construed as a potential conflict of interest.

## Publisher’s note

All claims expressed in this article are solely those of the authors and do not necessarily represent those of their affiliated organizations, or those of the publisher, the editors and the reviewers. Any product that may be evaluated in this article, or claim that may be made by its manufacturer, is not guaranteed or endorsed by the publisher.

## Footnotes

^1^http://www.genome.ucsc.edu/

^2^http://www.ensembl.org
