# Supplementary material for: Parkinson’s disease model in zebrafish using intraperitoneal MPTP injection
Source: Front Neurosci. 2023 Aug 25;17:1236049. doi: 10.3389/fnins.2023.1236049 (PMC10485380; doi:10.3389/fnins.2023.1236049)
Supplement: Supplementary file 1 [file Data_Sheet_1.PDF]

frontiers

Supplementary Material

Parkinson's Disease Model in Zebrafish using Intraperitoneal MPTP Injection

Noor Azzizah Omar, Jaya Kumar, Seong Lin Teoh*

*** Correspondence:** Dr Seong Lin Teoh: teohseonglin@ukm.edu.my

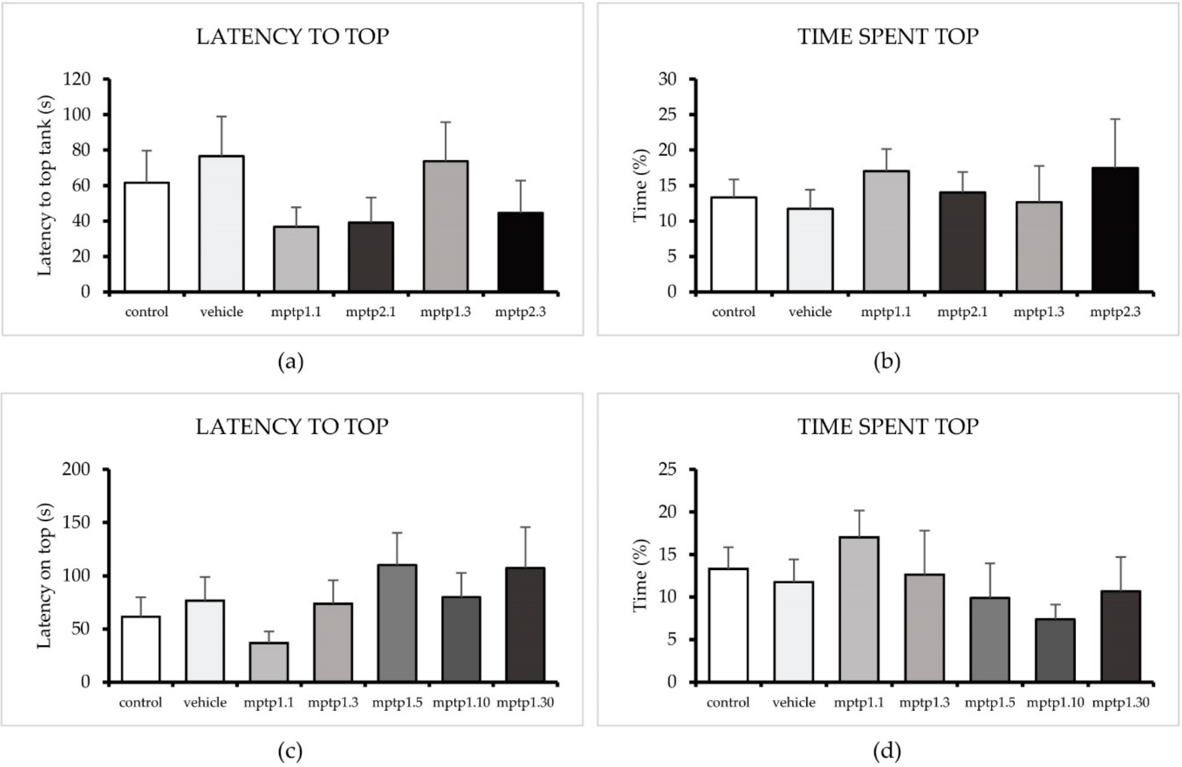

Supplementary Figure 1. Graph showing the time taken for the fish to reach the top tank and the mean percentage of time they spend in the top tank, (a) & (b): The differences in one- and two-times injection of MPTP. (c) & (d): The graph distribution from day one to 30 of MPTP groups control. There is some amount of increase in latency to reach the top and reduce the percentage of time spent on top tank in MPTP1.3 and MPTP1.5 however the results did not reach statistically significant result. All data are presented as mean ± SEM.

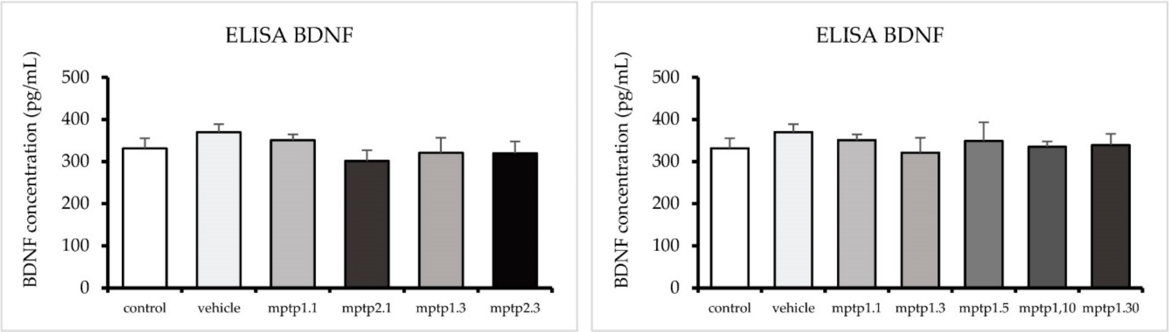

(a) (b)

Supplementary Figure 2. ELISA test for BDNF. BDNF level remains equivocal to control across the group in one- and two-time injections (a) and throughout the period of 30 days of assessment (b). All data is presented as mean ± SEM. Asterisks (*) indicate a significant difference between control fish and treated fish at *p<0.01 or **p<0.05.

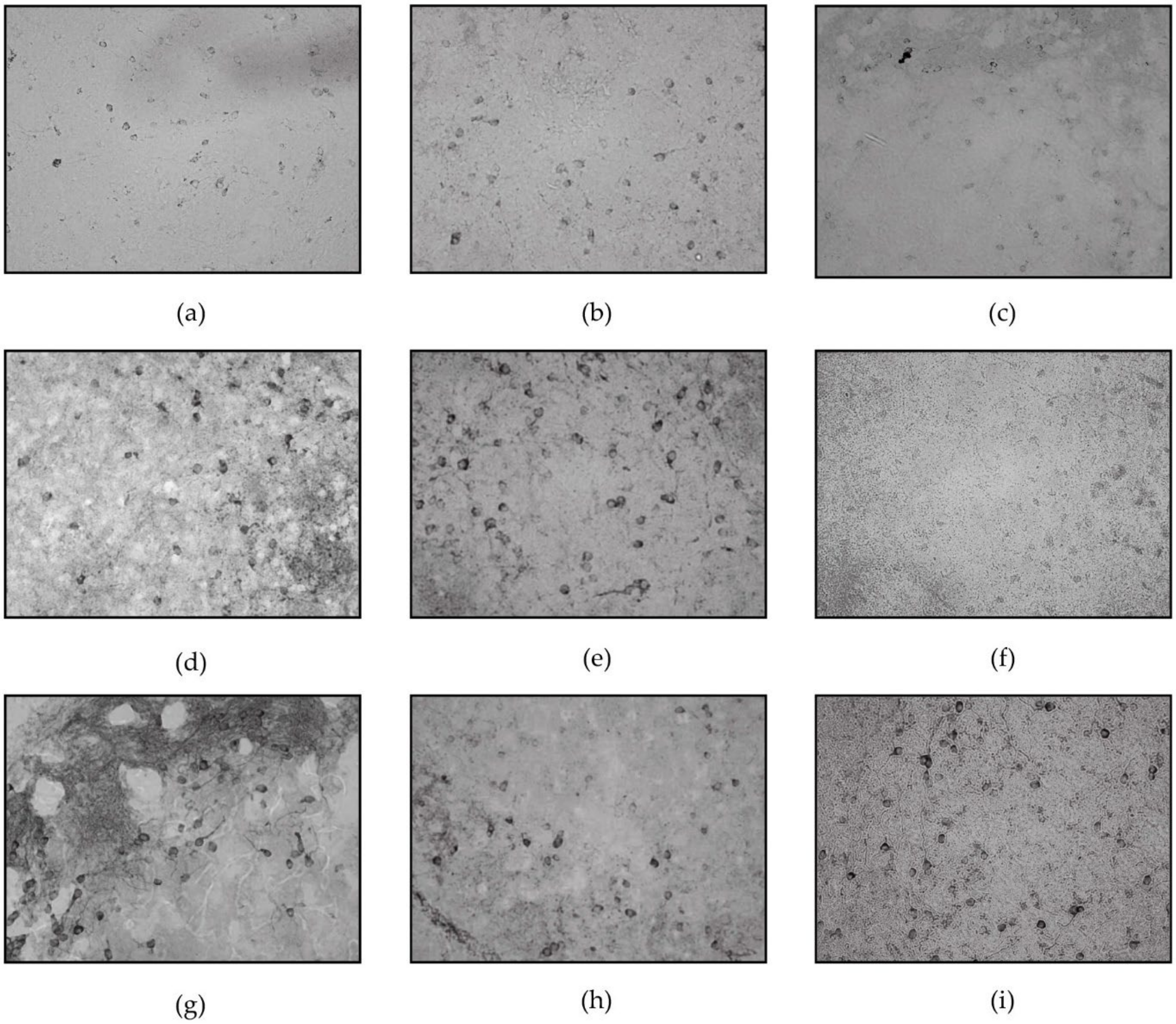

Supplementary Figure 3. Coronal sections of the olfactory bulb. (a): Control, (b): Vehicle, (c): MPTP1.1, (d): MPTP2.1, (e): MPTP1.3, (f): MPTP2.3, (g): MPTP1.5; (h): MPTP1.10, (i): MPTP1.30.

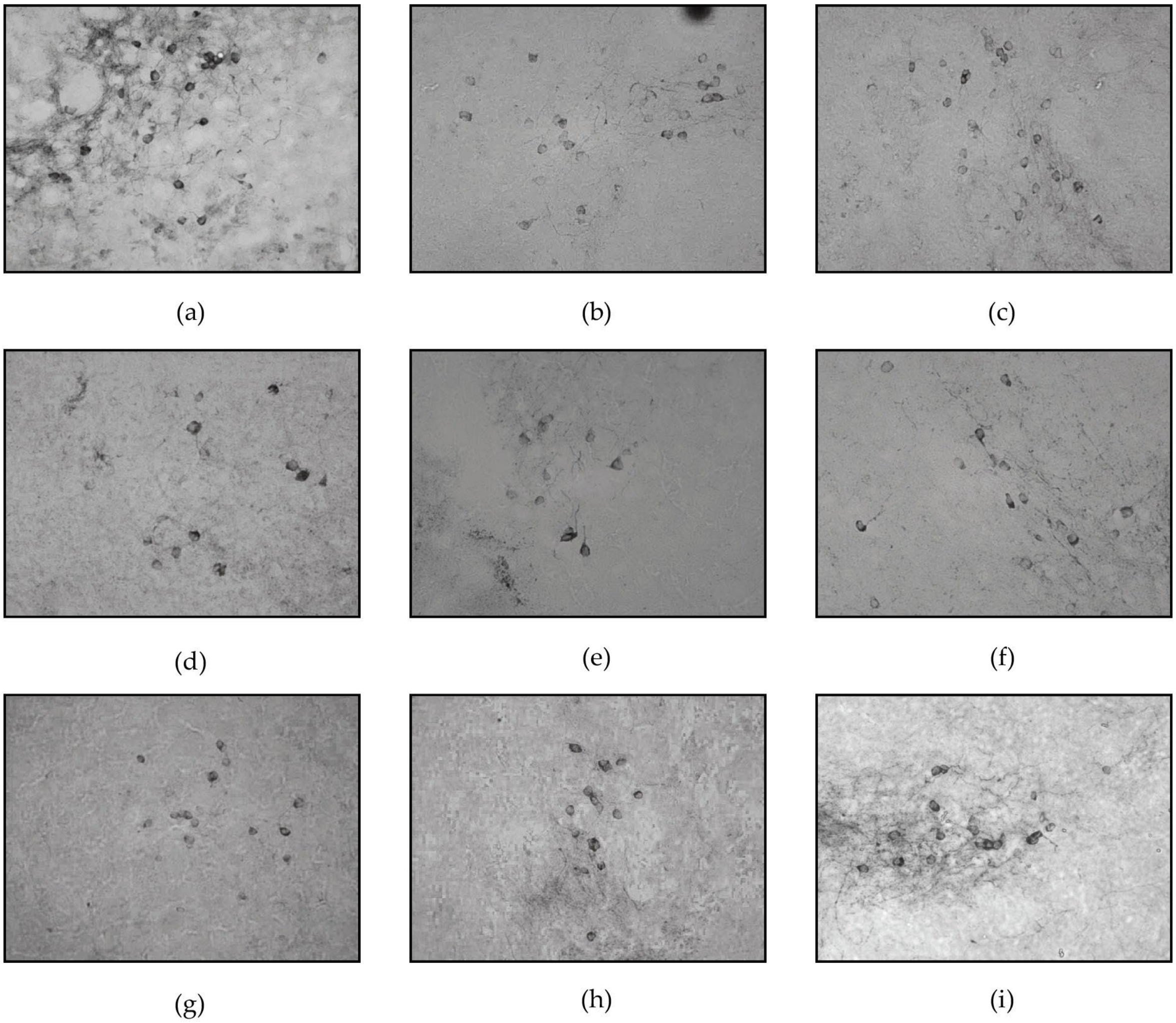

Supplementary Figure 4. Coronal sections of the subpallidum. (a): Control, (b): Vehicle, (c): MPTP1.1, (d): MPTP2.1, (e): MPTP1.3, (f): MPTP2.3, (g): MPTP1.5; (h): MPTP1.10, (i): MPTP1.30.

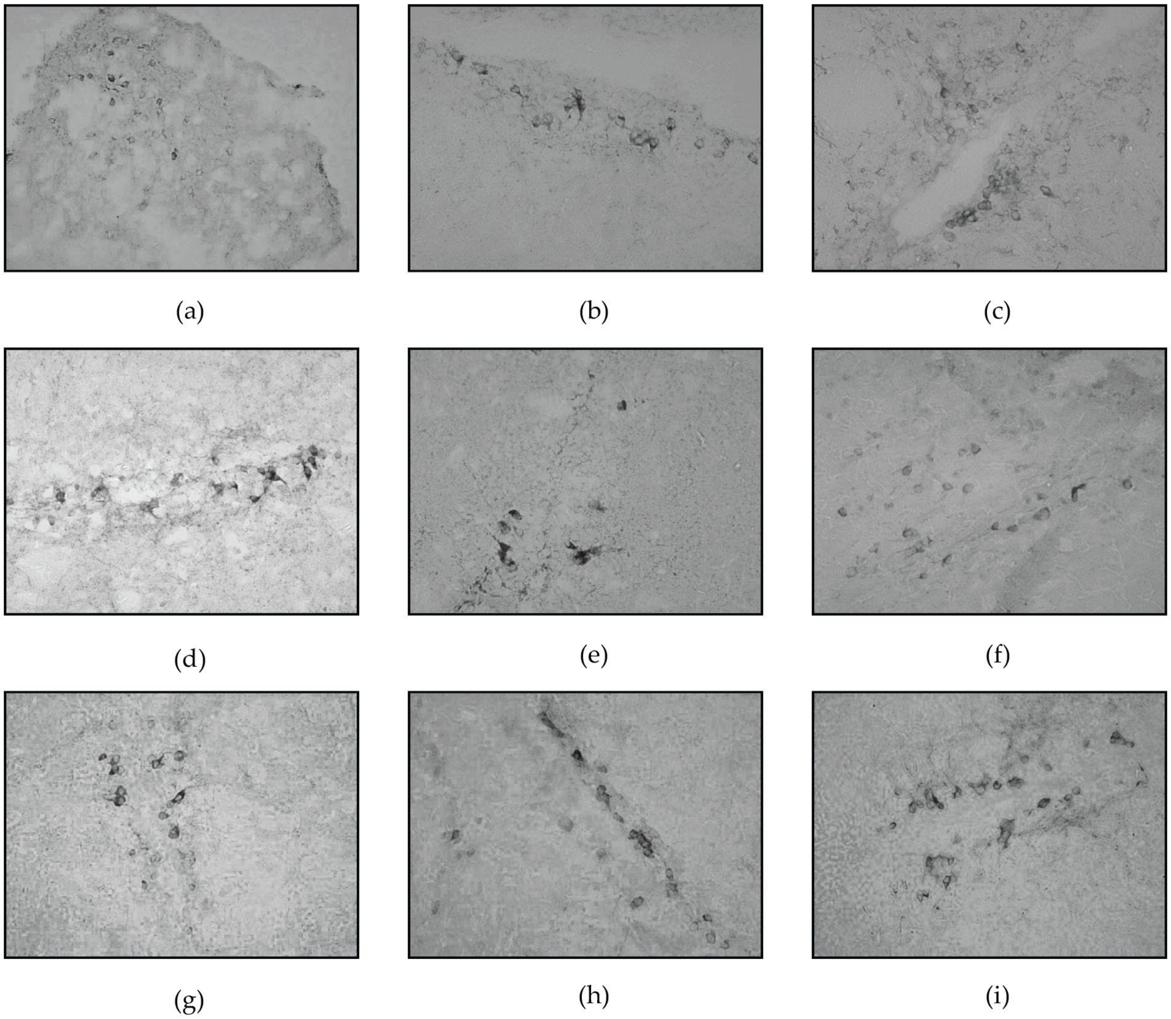

Supplementary Figure 5. Coronal sections of the preoptic region. TH+ cell counts declined on day 1 post MPTP for both one- and two-time injections. This persisted on day 3, the one-time injection group. However the remaining days, the TH+ cell counts were equivocal to the control. (a): Control, (b): Vehicle, (c): MPTP1.1, (d): MPTP2.1, (e): MPTP1.3, (f): MPTP2.3, (g): MPTP1.5; (h): MPTP1.10, (i): MPTP1.30.

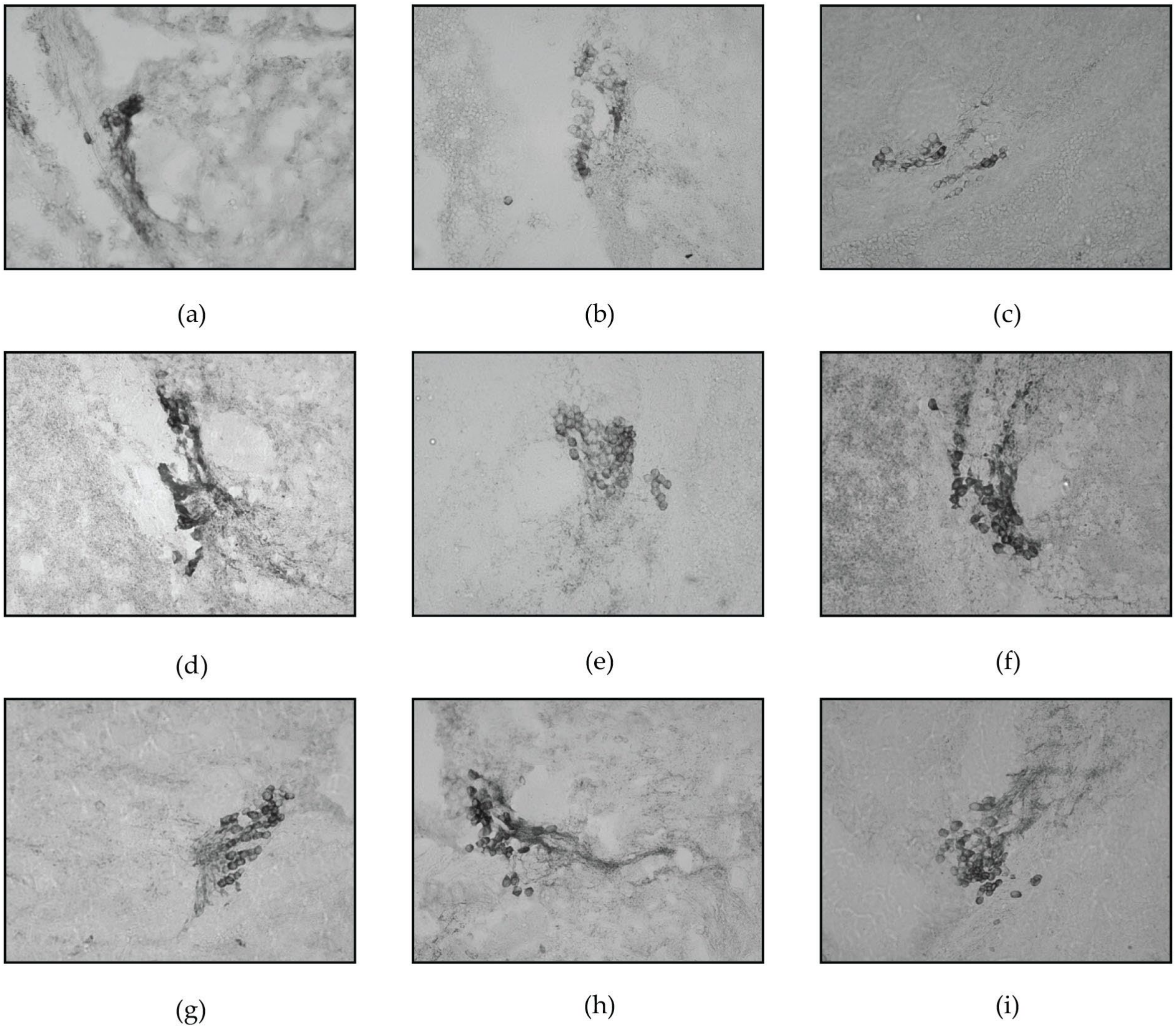

Supplementary Figure 6. Coronal sections of the pretectum region. TH+ cell counts were equivocal to the control except for day 30 which has shown a significant increment of dopaminergic neuron in this region. (a): Control, (b): Vehicle, (c): MPTP1.1, (d): MPTP2.1, (e): MPTP1.3, (f): MPTP2.3, (g): MPTP1.5; (h): MPTP1.10, (i): MPTP1.30.
